# Performance Study of High-Speed Permanent Magnet Synchronous Motor with Amorphous Alloy Considering Temperature Effect

**DOI:** 10.3390/ma17081928

**Published:** 2024-04-22

**Authors:** Changhao Yan, Haiyang Hu, Zhiye Li, Lubin Zeng, Ruilin Pei

**Affiliations:** 1 Department of Electric Engineering, Shenyang University of Technology, Shenyang 110178, China; yanchanghao@smail.sut.edu.cn (C.Y.);; 2 Suzhou Inn-Mag New Energy Ltd., Suzhou 215000, China

**Keywords:** amorphous alloy, permanent magnet synchronous motor, finite element simulation, iron loss factor, temperature

## Abstract

Because the magnetic properties of an amorphous alloy (AA) obviously change with the change of temperature, a finite element simulation method for a motor, considering the effect of temperature, is proposed in this paper. In the early design stage of the high-speed permanent magnet synchronous motor (PMSM), the simulation of motor performance is mainly based on the magnetic performance test data at room temperature provided by the material’s manufacturer. However, the influence of the temperature rise during the actual operation of the motor will lead to large errors between the simulation results and the measured results. Therefore, it is of great practical significance to measure the magnetic properties of the AA at different temperatures and use them for simulation purposes. In this paper, the magnetization characteristics and iron loss characteristics of the AA and silicon steel (ST100) used for comparison are measured at different temperatures, and the iron loss separation of the two materials at different temperatures is completed, and the hysteresis loss coefficient and eddy current loss coefficient at different temperatures are obtained. On this basis, the performance simulation of a motor model is carried out. The more accurate simulation method proposed in this paper can provide a reference for the design of AA motors in industry.

## 1. Introduction

With the continuous progress of society, the importance of developing energy-saving equipment, green economy and low-carbon industry is self-evident. In the field of motors, due to the advantages of high efficiency, high power density, lightweight nature and high control accuracy, the high-speed permanent magnet synchronous motor is gradually replacing the traditional AC asynchronous motor to become a new development trend in important industrial fields such as aerospace equipment, energy storage flywheels and precision machine tools [1,2,3]. However, in the working process of the high-speed permanent magnet synchronous motor, the high magnetization frequency caused by the high speed will lead to a large increase in the loss of the stator and rotor core, making the motor heat noticeably, which will lead to a reduction in the performance of the motor and lead to serious irreversible demagnetization of the permanent magnet or even damage to the casing, resulting in the substandard safety performance of the motor. As a new type of soft magnetic material, amorphous alloy (AA), has lower iron loss than other traditional soft magnetic materials [4,5]. This can solve the problem of the high iron loss of the high-speed permanent magnet synchronous motor under the high-frequency condition. At present, there are also many cases of the application of AAs in motors, In 2010, Hitachi of Japan produced an AA axial flux permanent magnet synchronous motor with rated power of 200 W and a rated speed of 3000 rpm. The stator of the motor is made by the winding method, so the adverse effect of processing technology on the magnetic properties of the AA is largely avoided [6]. Only one year later, Hitachi developed an AA slotless axial flux motor with rated power of 400 W and a rated speed of 15,000 r/min, which further improved the efficiency [7]. Reference [8] developed an AA motor with 8 poles and 24 slots. The measurement results show that compared with the traditional silicon steel motor, the iron loss of the AA motor is reduced by 80.28%. And the structure of AA motor is optimized by an evolutionary algorithm to further reduce the iron loss and copper loss. In reference [9], the iron loss of two different silicon steels (0.1 mm and 0.2 mm) and an AA was measured, and the iron loss of the AA was significantly smaller than that of the two types of silicon steel. The winding mode and rotor structure of the AA motor are improved, so as to further reduce the copper loss and mechanical loss. In the literature [10], Junyong Chen et al. made an AA PMSM and compared its simulation performance with a silicon steel PMSM with the same structure. It was found that although the cogs torque and torque of the two motors were almost the same, the iron loss of the AA motor was only 5% of that of the silicon steel motor. The measured results of the prototype also show the authenticity of the simulation and the feasibility of using an AA to make the motor. However, while AAs have excellent performance, they also have defects. For example, AAs are much more sensitive to temperature and stress than traditional silicon steel [11,12,13,14], and their mechanical properties, magnetization characteristics and iron loss characteristics will change greatly with the stress and ambient temperature they are in. Eventually, the performance of an AA PMSM will be adversely affected, such as the increase of noise and the decrease of efficiency [15,16,17]. In the motor, the stress mainly comes from assembly defects such as the interference of the shell and stator and rotor, and the residual stress inside the stator and rotor. The stress can be controlled by optimizing the motor assembly process or via post-processing measures such as annealing. However, the temperature rise in the electric machine when it works for a long time is difficult to suppress by measures similar to the control stress, and it more depends on the reasonable design of the internal parameters of the motor and the reasonable use of the motor control mode. Therefore, before designing an AA permanent magnet synchronous motor, it is necessary to fully consider the change in the magnetic properties of the AA and the change in motor performance caused by the temperature rise during the motor’s operation. At present, the research on the performance simulation analysis of permanent magnet synchronous motors is more based on the influence of the motor structure or the motor control mode on the motor performance. Moreover, the current research on the thermal simulation analysis of the motor is more focused on the motor simulation accuracy. For example, a 3D thermal network simulation model was proposed in the literature [18], and compared with the fluid-structure coupling finite element model, it was found that the errors of the proposed 3D thermal simulation model at the windings and stator cores were significantly smaller than those of the fluid-structure coupling model. But the error at the frame was much larger. Reference [19] coupled the numerical thermal model with the electromagnetic model and provided the influence of the inlet temperature and volume flow rate of the coolant at some load points in the motor on the heat transfer coefficient of the cooling tube, but it did not consider the influence of temperature on the magnetic properties of the material itself.

However, the research fully considering the influence of temperature on the magnetic properties of soft magnetic materials and combining it with the performance simulation of permanent magnet synchronous motors is still in its infancy. Therefore, this paper focuses on exploring the variation of the magnetic properties of an AA at different temperatures. The separation method of iron loss and the influence of temperature on the coefficient of the AA iron loss formula are introduced, which provide more accurate data support for the subsequent design and simulation of an AA permanent magnet synchronous motor. After that, the AA data measured at different temperatures and the iron loss separation coefficient are substituted into the finite element software to simulate the performance of the motor model and analyze the results. The same work is carried out on 0.1 mm thick silicon steel (ST100), so as to compare the sensitivity of the magnetization characteristics and iron loss characteristics of the AA and silicon steel to temperature, and the degree of change in the performance of the AA motor and ST100 motor with temperature.

In the second part of this paper, the device for variable temperature magnetic property testing and the selection of the magnetic property testing methods are introduced in detail. In the third part, the influence of temperature on the magnetic properties of the AA is analyzed, and then the magnetic properties of the AA and ST100 are compared and analyzed, and the separation of the consumption coefficient of the AA and ST100 under different temperature and frequency test conditions is completed. The fourth part introduces the motor model and its basic parameters, and it imports the magnetic properties of the two materials obtained in the third part into the motor model, and finally, it simulates and compares the performance of the AA motor and ST100 motor at different temperatures.

## 2. Experimental Equipment and Sample Preparation

**Figure 1:**
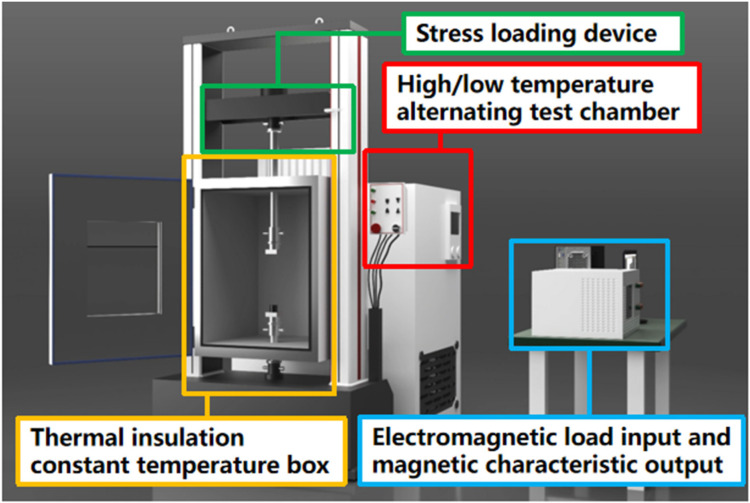
Multi-physical field testing equipment for magnetic properties of soft magnetic materials.

As shown in Figure 1, the multi-physics field test system used for magnetic property testing in this paper is coupled with a stress loading module, a high- and low-temperature loading module, and a frequency conversion magnetic property testing module. In this experiment, a high- and low-temperature loading module and a frequency conversion magnetic properties test module are used. The iron loss and magnetization characteristics of the AA and ST100 samples under a multi-physics coupling environment with variable temperature and frequency are measured.

**Figure 2:**
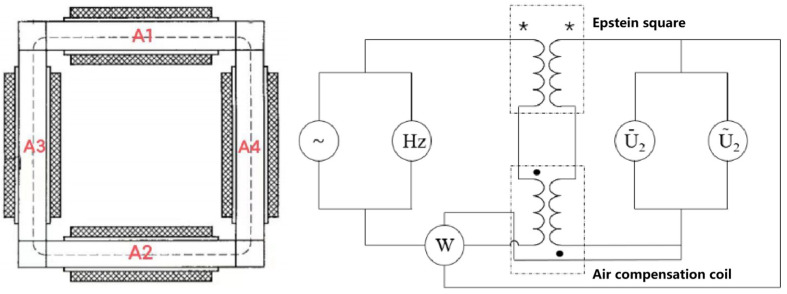
The principle of Epstein’s square circle method.

At present, the commonly used methods for magnetic property testing include Epstein’s square circle method, the monolithic method and the ring sample method. The most popular method is Epstein’s square circle method. Figure 2 shows the principle of Epstein’s square circle method. The test principle of Epstein’s square circle is similar to that of a no-load transformer, which contains four groups of coils, and each group of coils has an excitation winding and a measurement winding, which are connected in series, respectively, In Figure 2, the dotted terminal of the excitation winding and the measurement winding of the square coil are denoted by *. At the same time, in order to reduce the influence of air between windings on the measurement results, the air compensation winding are reversed in series, and the dotted terminal of the air compensation winding are represented by black dots in Figure 2. When a certain frequency of AC voltage is applied to the excitation winding, the induced voltage will be generated in the measurement winding. By detecting the magnitude of the induced voltage, the magnetic properties of the measured material, such as the magnetic flux density and iron loss, can be calculated. During the test, in the form of a double lap, the silicon steel sheet in the rolling direction is placed in A1 and A2 for testing, and the silicon steel sheet perpendicular to the rolling direction is placed in A3 and A4 for testing. And then, a square closed magnetic circuit is formed. The principle of the magnetic property testing of the monolithic method is similar to that of the Epstein method, so this manuscript will not discuss it. The test samples for both testing methods are in the form of monolithic, and the magnetic circuit is not circular.

**Figure 3:**
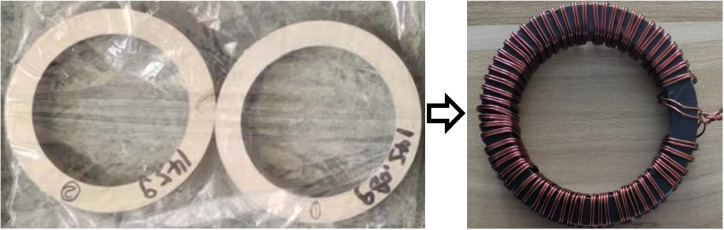
Test samples of the magnetic properties of AA and ST100.

But, as the stator and rotor in the motor are core rather than sheet, and the stator and rotor of the motor are mostly round rather than square, the test results of Epstein’s square circle method and the monolithic method will deviate from the magnetic properties of the motor during actual operation, while the ring sample method has the advantages of a uniform magnetic circuit, sample shape close to the stator and rotor core, easy to apply temperature, small fluctuation and stable performance, which the other two methods do not have [20]. Therefore, this paper chooses the ring-like method as the method for magnetic property testing. Figure 3 shows standard ring samples of the AA and ST100, which have an outer diameter of 60 mm, an inner diameter of 40 mm, and a height of 10 mm. All of them are made of multi-layer bonded laminates, and the interlayer adhesive is a self-adhesive organic coating with good insulation and stability. After the preparation of the ring sample, the copper wire is used to wind the excitation winding and the measurement winding, where the excitation winding is 100 turns and the measurement winding is 15 turns. (Refer to GB/T 3658-2022 for the method of making the ring samples in this manuscript).

In this experimental device, the temperature adjustment inside the insulation box is realized through the thermal radiation equipment and the coolant channel. During the heating process, the thermal radiation equipment can achieve zero contact with the experimental ring sample, so as to ensure the uniform heating of the ring sample as a whole. In the cooling process, rapid cooling can be realized through the coolant in the flow channel. Finally, the internal temperature of the box is adjusted by the built-in high-precision and high-sensitivity temperature sensor, and the real-time tracking and precise control of the internal temperature of the box is realized. At the same time, the electrical steel broadband magnetic characteristic measuring instrument is used as the electromagnetic module. The two ends of the ring-like excitation winding and the two ends of the measuring winding are correctly connected to the measuring instrument during the experiment, and the corresponding magnetic flux density or magnetic field strength test points are input into the host computer (such as 0.1 T, 0.2 T, 1000 A/m). The device can control the size of the excitation field by applying different voltages and currents to the excitation winding of the sample, and performing real-time detection of the voltage and current at the measurement winding end. Finally, according to the internal algorithm of the device, the hysteresis loop of different test points and magnetic performance data such as the iron loss and magnetic flux density can be obtained. In summary, the magnetic properties of ring samples in a variable temperature field and variable frequency field can be tested with this kind of device.

The experimental steps in this manuscript are as follows. Firstly, the temperature of the high/low-temperature alternating test chamber is set after the test ring sample is placed in the high/low-temperature alternating test chamber. When the temperature of the high/low-temperature alternating test chamber reaches the setting point, the heat preservation is carried out. In order to make the temperature of the sample consistent with the temperature of the high/low-temperature alternating test chamber, the heat preservation time is controlled at about one hour. After that, the primary winding and secondary winding of the test sample are, respectively, connected to the AC magnetic property test system (the device in the lower right corner of Figure 1), and different test conditions such as the frequency, magnetic field strength and magnetic flux density are input into the host computer, which is connected to the AC magnetic property test system. Finally, the iron loss characteristics and magnetization characteristics of the sample at different temperatures and frequencies can be measured.

## 3. Experimental Results: Performance Curve

The working temperature is an important factor to be considered in the process of motor design. Different soft magnetic materials have different sensitivity to temperature. A lack of research on the influence of temperature on the magnetic properties of soft magnetic materials may cause adverse situations in the actual work of the motor. Therefore, a temperature change experiment involving soft magnetic materials is a necessary prerequisite for the design of a high-reliability motor. This section mainly tests and analyzes the magnetic properties of the AA at different temperatures, separates the losses according to the iron loss curve, and analyzes the changes in the iron loss coefficient of the AA at different temperatures and different frequencies. Since the thickness of the AA is much lower than that of ordinary silicon steel, for the sake of the comparability of the experimental objects, this paper chooses the lowest thickness (0.1 mm) extremely thin silicon steel (ST100) on the market to set up the experimental control group. The test conditions, test equipment and test methods of the two materials are the same.

### 3.1. Test Results and Analysis of Magnetic Properties of AA

**Figure 4:**
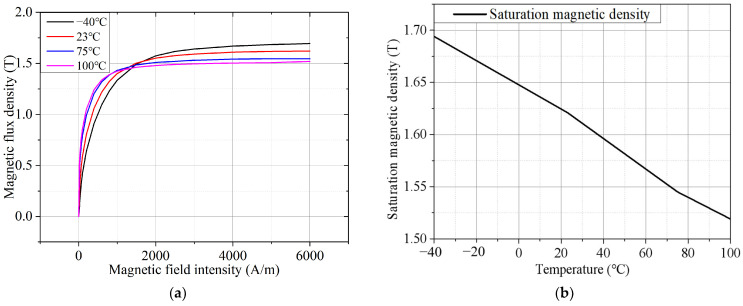
Magnetization characteristics of AA at different temperatures (frequency = 50 Hz): (**a**) B–H curve; and (**b**) saturation flux density of AA at different temperatures.

The B–H curves of the AA at different temperatures when the test frequency is 50 Hz are shown in Figure 4a. The magnetization curves at different temperatures almost intersect when the magnetic field strength is about 1300 A/m to about 1500 A/m, that is, the magnetic flux density generated under the same magnetic field strength is almost the same. When the magnetic field strength is significantly lower than 1300 A/m, with the increase in the temperature, the magnetic permeability of the AA will increase, so the magnetic flux density of the AA will increase with the increase in temperature under the same magnetic field strength. On the contrary, if the magnetic field strength is significantly greater than 1500 A/m, the magnetic flux density of the AA under the same magnetic field strength will show a decreasing trend with the increase in temperature. When the temperature increases from −40 °C to 100 °C, the saturation magnetic density of the AA decreases from 1.694 T to 1.519 T, a decrease of 10.33%. It is easy to see from Figure 4b that the saturation magnetic density of the AA is inversely proportional to the temperature and approximately linear, that is, for every 20 °C increase in temperature, the saturation magnetic density decreases by about 0.025 T.

**Figure 5:**
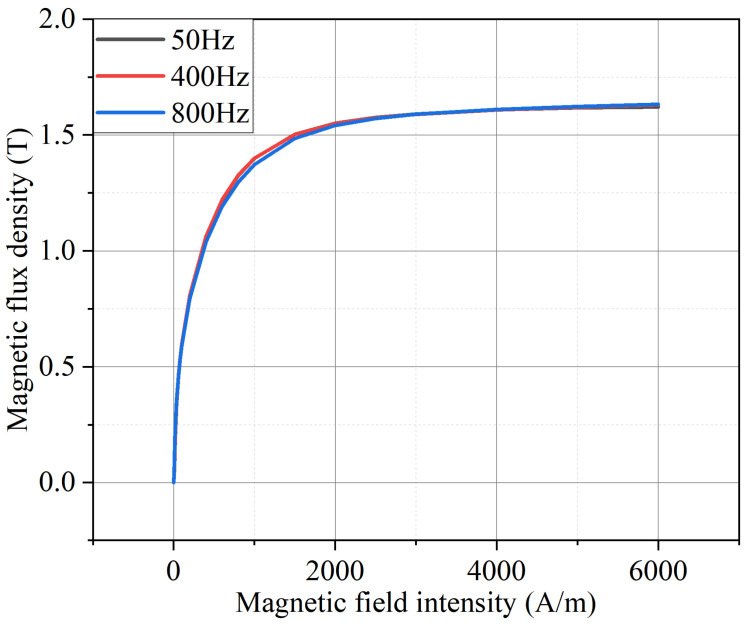
Magnetization characteristics of AA at different frequencies (temperature = 23 °C).

In order to explore the influence of frequency on the magnetization characteristics of the AA, this manuscript provides the magnetization curves at different frequencies when the temperature is 23 °C, and the results are shown in Figure 5. It can be seen that the frequency has little effect on the magnetization characteristics of the AA, and the degree of the AA magnetization curve changing with the frequency is far less than that changing with the temperature. When the frequency is increased from 50 Hz to 800 Hz, the saturation magnetic density of the AA is only increased from 1.621 T to 1.632 T, an increase of about 0.67%. Therefore, the effect of frequency on the AA magnetization curve can be chosen to be ignored when designing the AA motor.

**Figure 6:**
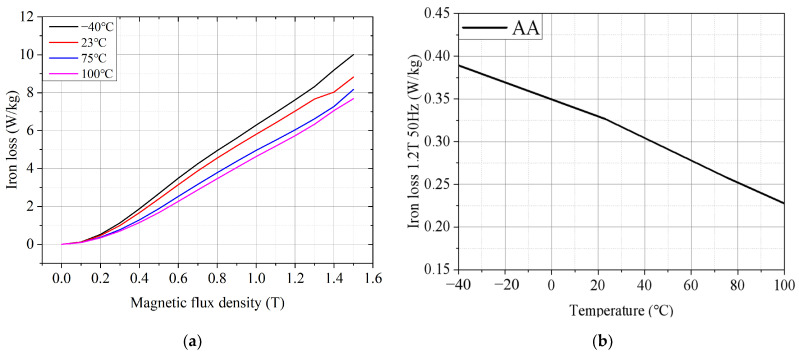
Iron loss characteristics of AA at different temperatures: (**a**) B–P curve(frequency = 800 Hz); and (**b**) iron loss of AA at different temperatures (frequency = 50 Hz, magnetic flux density = 1.2 T).

Figure 6a shows the test results of the iron loss of the AA at different temperatures at the frequency of 800 Hz. It is easy to see that when the magnetic flux density is the same, the iron loss of the AA decreases with the increase in temperature. The main reason is that the resistivity of the AA increases with the increase in temperature, and the eddy current generated under the same induced electromotive force is small, so the eddy current loss will be reduced. Figure 6b shows the test results of the iron loss of the AA at different temperatures when the frequency is 50 Hz and the magnetic flux density is 1.2 T. It can be seen that the change trend of the iron loss of the AA at different temperatures is similar to the change trend of the saturated magnetic density, both of which are inversely proportional to the temperature and approximately linear.

### 3.2. Comparative Analysis of Magnetic Properties between AA and ST100

**Figure 7:**
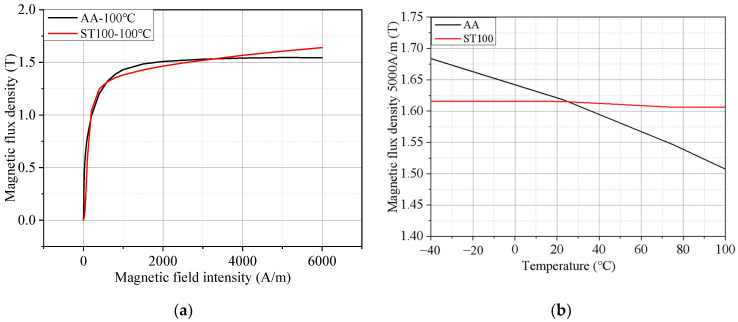
Magnetization properties of AA and ST100 (frequency = 50 Hz): (**a**) B–H curve; and (**b**) magnetic flux density of AA and ST100 at different temperatures (magnetic field intensity = 5000 A/m).

The measured temperature rise of the motor used in this paper in the rated operation is 106.4 °C. Therefore, the magnetization curve test data of the two materials at a temperature of 100 °C were selected for comparison. Figure 7a shows the B–H curves of the AA and ST100 at 100 °C. It is easy to see that the magnetic density of the AA is higher than that of the ST100 under the same magnetic field intensity before the magnetic field intensity is about 2500 A/m, and higher magnetic density can bring higher torque to the motor. Moreover, the magnetic permeability of the AA is also significantly higher than that of the ST100. However, after the magnetic field intensity is 2500 A/m, the AA has deep magnetic saturation at this stage, and because the saturation magnetic density (1.52 T) of the AA at 100 °C is only 90.48% of the saturation magnetic density (1.68 T) of the ST100, the magnetic density of the AA is lower than that of the ST100 at the same magnetic field intensity. Therefore, in the design of an AA permanent magnet synchronous motor, special attention should be paid to the adverse effects of the low magnetic density and low magnetic permeability caused by the magnetic saturation of the AA, and the magnetic density inside the core should not be higher than 1.49 T (corresponding to a magnetic field strength of 2500 A/m) under various working conditions. Figure 7b shows the magnetic flux density of the AA and ST100 at different temperatures when the test frequency is 400 Hz and the magnetic field intensity is 5000 A/m. It is easy to see that compared with the ST100, the magnetization characteristics of the AA are more sensitive to the temperature. When the temperature rises from −40 °C to 100 °C, the magnetic flux density of the ST100 decreases by only 0.6%, which is far lower than the 10.3% of the AA. This is due to the fact that the AA has a different microstructure to the ST100. AAs have amorphous atomic arrangements due to their grain-free nature. The silicon steel has a grain structure and a grain-ordered arrangement. The amorphous structure of the AA makes it subject to stronger thermal excitation and thermal fluctuation at high temperatures, which leads to the rearrangement of the magnetic domains and the difficulty of maintaining magnetization. Finally, the deterioration of the magnetic sensing characteristics of the AA at high temperatures is much greater than that of ordinary silicon steel, which may lead to the substandard magnetic flux density of the motor and affect the torque. Therefore, it is very important to consider the influence of temperature on the magnetic density characteristics when designing motors with Aas.

**Figure 8:**
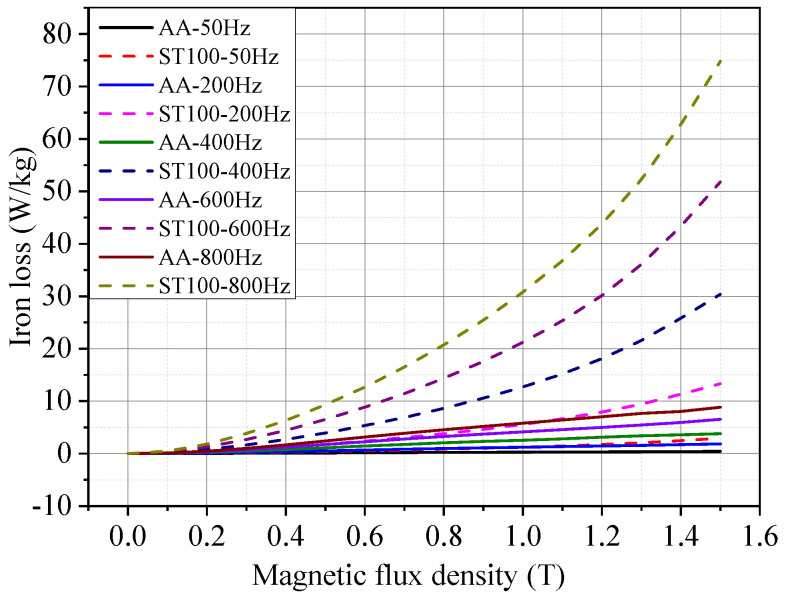
Iron loss of AA and ST100 at different test frequencies (temperature = 23 °C).

Figure 8 shows the iron loss test results of the AA and ST100 at 23 °C at different frequencies. It can be seen that the iron loss of the AA is much less than that of the ST100 at the same frequency, and the absolute value of the iron loss difference between the two increases with the increase in frequency and magnetic flux density. As can be seen from Table 1, in terms of the multiple, the iron loss of the ST100 at a low frequency (50 Hz, 1.0 T, for example) is 4.32 times that of the AA, and the iron loss of the ST100 at a high frequency (800 Hz, 1.0 T, for example) is 5.29 times that of the AA. Therefore, the higher the frequency, the more obvious the advantage of the low iron loss of the AA. The reason is mainly because there is no regular crystal structure and no obvious grain boundary in the AA, which makes the magnetic domain in the AA move and rotate freely, so that its hysteresis loss is lower than that of the ST100 material. On the other hand, the thickness of the AA is only about 0.03 mm, far lower than that of the ST100 (0.1 mm). This makes the eddy current loss of the AA also much smaller than that of the ST100.

**Figure 9:**
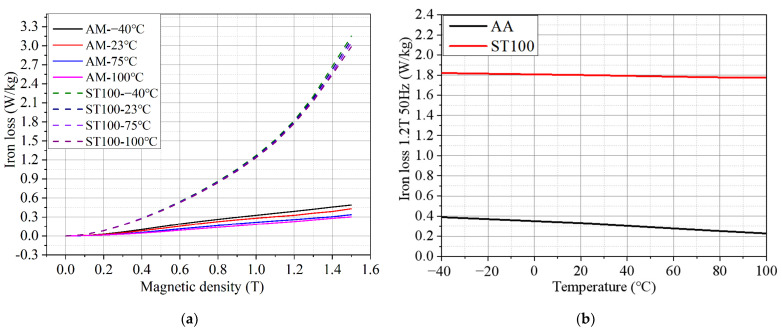
Iron loss of AA and ST100 at different temperatures (frequency = 50 Hz): (**a**) B–P curve; and (**b**) iron loss of AA and ST100 at different temperatures (magnetic flux density = 1.2 T).

Figure 9a shows the iron loss comparison of the ST100 and AA at different temperatures when the test frequency is 50 Hz. It can be seen from Figure 9a that, taking the test point with a magnetic flux density of 1.5 T as an example, when the temperature rises from −40 °C to 100 °C, the iron loss of the ST100 decreases from 3.152 W/kg to 2.986 W/kg. The iron loss of the AA is reduced from 0.490 W/kg to 0.302 W/kg, which is reduced by 38.37%. Figure 9b shows the iron loss of the ST100 and AA at different temperatures when the test frequency is 50 Hz and the magnetic flux density is 1.2 T. It can be more intuitively seen from Figure 9b that the iron loss of the AA has an approximately linear relationship with the temperature, which is similar to the law of magnetic flux density and temperature change of the AA described in Figure 8b above. At the same time, by observing the respective slopes of the two curves, it is easy to see that the iron loss of the AA is much more sensitive to the temperature change than that of the ST100. This is because the magnetic domain structure of the AA is more complex and unstable, which can be easily rearranged and changed by temperature change, resulting in a large decrease in iron loss with a temperature increase. However, the magnetic domain structure of the ST100 is orderly and stable, and it is not relatively easy to change under the influence of temperature, so the change degree of iron loss with the increase in temperature is small. In addition, the accuracy of the iron loss test results of soft magnetic materials will directly affect the accuracy of the motor efficiency simulation and temperature rise simulation in the process of motor design. Therefore, in the process of designing an AA permanent magnet synchronous motor, in order to accurately determine the core loss and efficiency of the motor in the actual operation process, it is also very important to consider the influence of the temperature change on the iron loss characteristics of the AA.

### 3.3. Iron Loss Coefficient Separation

In order to compare the hysteresis loss coefficient and eddy current loss coefficient as a function of the temperature and frequency, in this manuscript, the iron loss coefficient is separated from the iron loss test results. The iron loss per unit weight can be calculated according to Bertotti’s loss separation model. According to previous studies, the value of the additional loss coefficient in the traditional trinomial iron loss model is generally lower than the hysteresis loss coefficient and the eddy current loss coefficient by more than two orders of magnitude [14], and its calculation requires a lot of work. Therefore, this manuscript chooses to ignore the additional loss coefficient and adopt Bertotti’s iron loss model in binomial form:

(1) $$P_{S}=K_{h}B^{\alpha }f+K_{c}B^{2}f^{2}$$

where *K_h_* represents the hysteresis loss coefficient and *K_c_* represents the eddy current loss coefficient. *α* is the iron loss coefficient, which usually takes a value between 1.5 and 2.0; in this paper, *α* is taken to be 1.6. *B* is the magnetic flux density. *f* is the frequency. In this paper, the hysteresis loss coefficient and eddy current loss coefficient of the AA and ST100 are obtained by using the Curve Fitting Tool (cftool) in Matlab R2021b. Table 2 shows the changes in the hysteresis loss coefficient and eddy current loss coefficient of the AA and ST100 at different test frequencies (temperature = 100 °C). It can be seen that the hysteresis loss coefficient and eddy current loss coefficient of the ST100 are much higher than those of the AA. This conclusion can be supported by the conclusion drawn in the previous section that the iron loss of the AA is much lower than that of the ST100. In particular, the gap between the eddy current loss coefficients of the two materials is up to two orders of magnitude. This is because the thickness of the AA is only about 1/3 that of the ST100. The resistivity of the AA is also higher than that of the ST100, so it has a lower eddy current loss coefficient. At the same time, the hysteresis loss coefficient of the ST100 almost does not change with the frequency change, and the hysteresis loss coefficient of the AA shows an upward trend with the increase in frequency. However, the eddy current loss coefficient of the AA and ST100 decreases with the increase in frequency. This further indicates that the sensitivity of the AA to external factors is greater than that of the ST100 material. The greater the working frequency of the motor, the greater the speed; therefore, when designing an AA motor, special attention should be paid to the influence of motor speed on the AA itself.

Table 3 shows the changes in the *K_h_* and *K_c_* of the AA and ST100 at different temperatures (frequency = 50 Hz). When the temperature changes, the hysteresis loss coefficient of the AA and ST100 fluctuates in a small range, and the eddy current loss coefficient decreases with the increase in temperature. However, the hysteresis loss coefficient of the AA decreases much more than that of the ST100 with the increase in temperature. After the temperature rises from −40 °C to 100 °C, the eddy current loss coefficient of the ST100 is only reduced by 8.27%, while the hysteresis loss coefficient of the AA is reduced by 93.11%. Therefore, the temperature has a great influence on the loss of the AA. The effect of temperature cannot be ignored in the application of the AA. The data fitting results are in good agreement with the previous experimental results that the iron loss of the AA decreases significantly with the increase in temperature.

**Figure 10:**
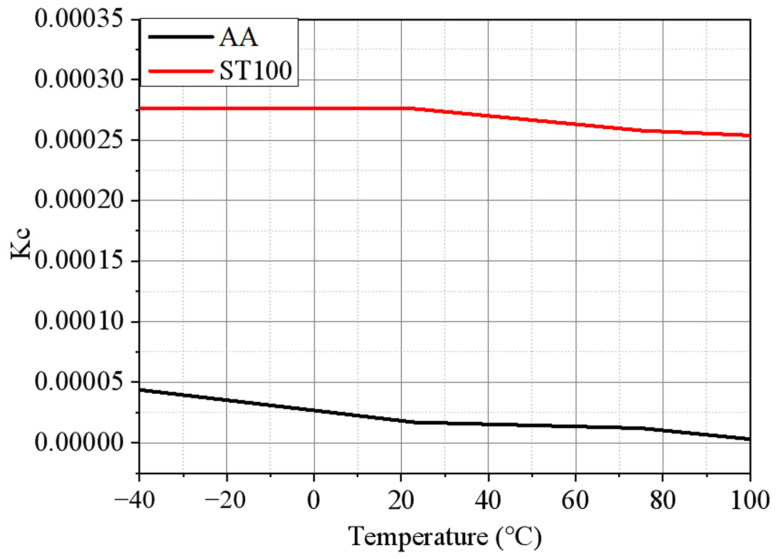
Eddy current loss coefficient of AA and ST100 at different temperatures (frequency = 50 Hz).

In Figure 10, the variation trend of the eddy current loss coefficient of the two materials at different temperatures can be seen more intuitively. It is easy to see that the eddy current loss coefficient of the ST100 changes very little with the temperature before about 40 °C, and decreases with the increase in temperature after 40 °C. The eddy current loss coefficient of the AA decreases rapidly with the increase in temperature in the whole temperature interval. The relationship between the eddy current coefficient of the AA and the temperature is similar to a linear negative correlation, and the eddy current loss coefficient of the AA decreases about 13.30% when the temperature increases by 20 °C. Therefore, from the extent to which the eddy current loss coefficients of the two materials change with the temperature, it can be seen that the temperature sensitivity of the iron loss characteristics of the AA is much greater than that of the ST100, which is mutually consistent with the conclusion drawn in the previous section.

## 4. Finite Element Model and Motor Performance Analysis

In order to compare the performance of the AA as a stator of high-speed PMSM under different temperature conditions, the performances of the AA stator PMSM and ST100 stator PMSM are calculated and analyzed using finite element simulation software in this section.

### 4.1. Motor Structure and Main Parameters

**Figure 11:**
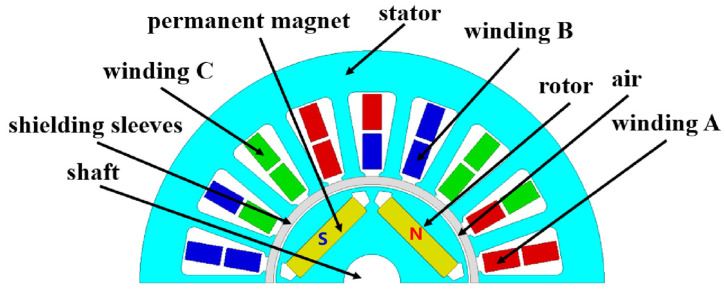
Finite element model of permanent magnet synchronous motor.

Figure 11 shows the PMSM model proposed in this paper. The pole slot ratio of the motor is 4 poles and 18 slots. The rotor part of the motor adopts a built-in structure. Because the processing of the AA is difficult, the rotor material is B25AHV1300, and the stator part is AA or ST100. The magnetic field line direction of the N-pole magnetic steel is the direction of the magnetic steel pointing to the winding, while the magnetic field line direction of the S-pole magnetic steel is the direction of the winding pointing to the magnetic steel. After passing through the stator and rotor core and winding in the middle, a closed magnetic circuit is formed. Because of the special use of this motor, a shielding cover should be added between the stator and the rotor to isolate the stator from the air gap. The rated speed of the motor is 21,000 rpm and the peak speed is 25,000 rpm. The main performance parameters of the motor are shown in Table 4.

### 4.2. Thermal Simulation Analysis of Motor

**Figure 12:**
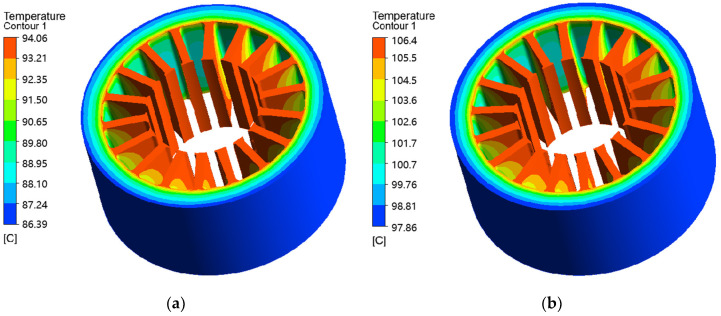

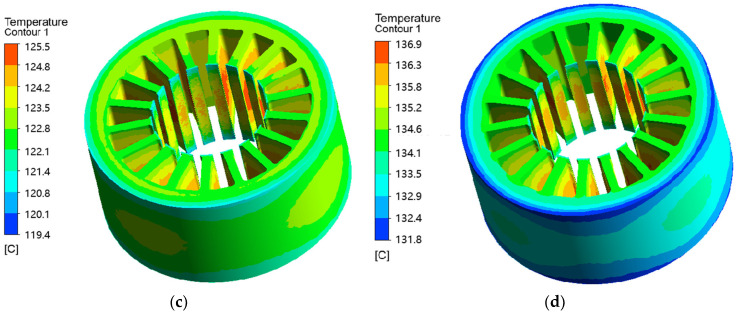
Temperature rise cloud image of AA and ST100 motors: (**a**) AA-no iteration; (**b**) AA-iteration; (**c**) ST100-no iteration; and (**d**) ST100-iteration.

Figure 12 shows the thermal simulation results of the permanent magnet synchronous motor in this paper. Since the material replacement in this paper is aimed at the stator, only the thermal simulation work of the motor stator is carried out. Because the iron consumption of the AA and ST100 is easily affected by the temperature, the iron consumption and temperature rise are coupled. The coupling relationship makes the thermal simulation of motor involve the iterative treatment of the material properties and motor temperature rise, which increases the difficulty of the motor thermal simulation. The Fluent module of the Ansys finite element simulation software supports the iterative thermal simulation function of the temperature rise and material properties. Therefore, the Fluent module of Ansys is used in this paper for the thermal simulation work of the AA motor and ST100 motor. As can be seen from Figure 12, the AA motor and ST100 motor both produce a very high temperature rise during operation, and there is a huge gap between the iterative and non-iterative temperature rise simulation results. The maximum temperature rise after the iteration of the ST100 motor is 11.4 °C higher than that without iteration, while the maximum temperature rise after the iteration of the AA motor is 11.6 °C higher than that without iteration. Moreover, by comparing the temperature rise of the AA motor and ST100 motor, it can be found that the highest temperature of the AA motor after the iterative thermal simulation is 106.4 °C, which is lower than the 136.9 °C of the ST100 motor. This not only validates the conclusion in Section 3 that the iron loss of the AA is much lower than that of the ST100 but also indicates that the influence and importance of iterative processing in the thermal simulation of the AA motor is greater.

### 4.3. Electromagnetic Simulation Analysis of Motor

The thermal simulation results of the AA motor in Section 4.2 show that the overall temperature rise of the AA motor is close to 100 °C, and the magnetic properties of soft magnetic materials used in the motor design are usually measured by the manufacturer under normal temperature conditions. Therefore, this section only selects the electromagnetic performance simulation of the rated operating condition of the motor at room temperature (23 °C) and 100 °C for comparison.

**Figure 13:**
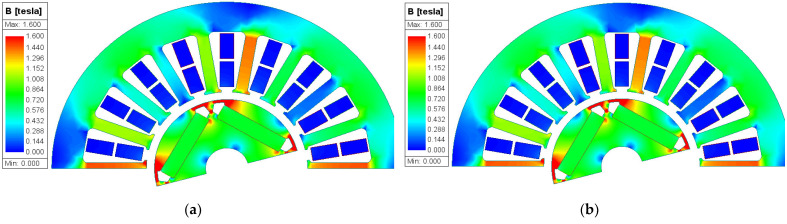

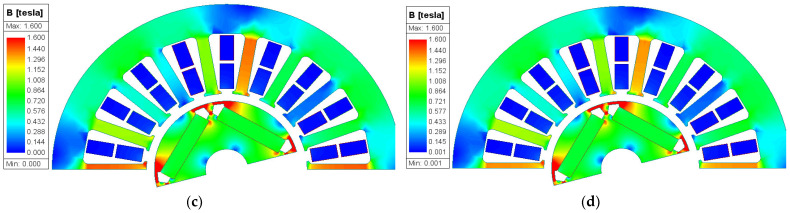
Magnetic flux density distribution of AA and ST100 motors at different temperatures: (**a**) ST100-23 °C; (**b**) ST100-100 °C; (**c**) AA-23 °C; and (**d**) AA-100 °C.

Figure 13 shows the magnetic flux density distribution of the ST100 motor and AA motor at 23 °C and 100 °C, and the material magnetic property data of the motor model at each temperature are the data measured at the corresponding temperature in Section 3. The results show that the magnetic flux density of the ST100 motor almost does not change with the change in temperature, and it has good temperature stability. However, it can be seen from the magnetic flux density distribution of the AA motor that the magnetic density of the teeth at 23 °C is greater than that at 100 °C. This mutually supports the conclusion in Section 3 that the temperature sensitivity of the magnetic flux density of the AA is greater than that of the ST100. It also shows that the effect of temperature on the magnetization of the AA cannot be ignored in the process of AA motor design.

**Figure 14:**
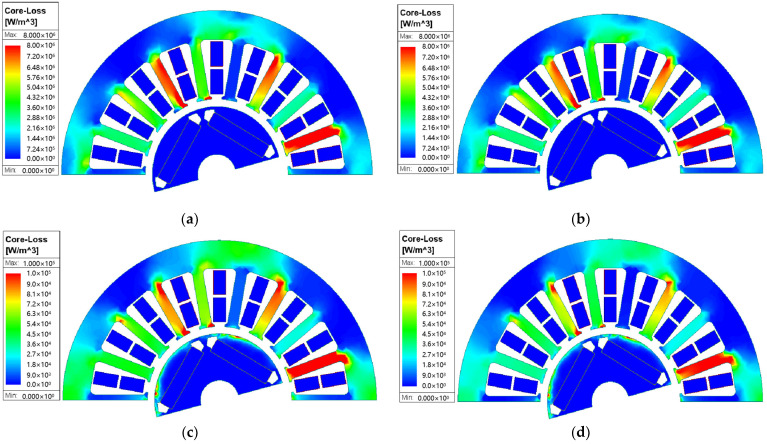
Iron loss distribution of AA and ST100 motors at different temperatures: (**a**) ST100-23 °C; (**b**) ST100-100 °C; (**c**) AA-23 °C; and (**d**) AA-100 °C.

Figure 14 shows the iron loss distribution of the ST100 motor and AA motor stator at 23 °C and 100 °C. It can be seen from the figure that the motor stator iron loss of the two materials is mainly concentrated in the teeth, which is caused by the high magnetic density of the motor teeth. Moreover, with the increase in temperature, the stator iron loss of the two materials decreases. However, the degree of iron loss of the AA stator changing with the temperature is still larger than that of the ST100 stator, which is consistent with the iron loss test results of the two materials at different temperatures in Section 3. The iron loss of the stator part of the motor will directly determine the temperature rise of the motor when it works. Therefore, the influence of temperature on the iron loss of the AA must not be ignored when simulating the efficiency and temperature rise of the AA permanent magnet synchronous motor; otherwise, the results will be divorced from reality and not accurate enough.

**Figure 15:**
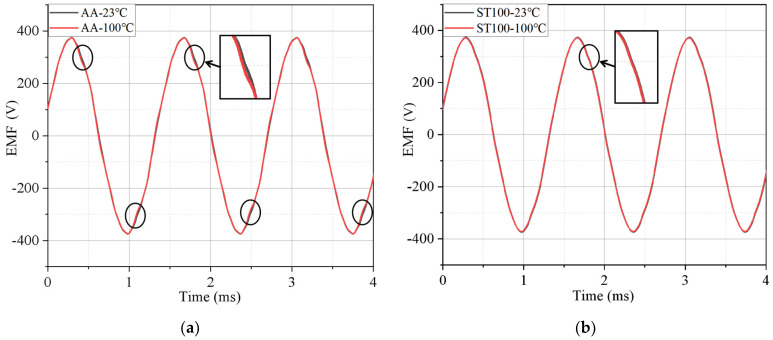
Back EMFs of AA and ST100 motors at different temperatures: (**a**) AA; and (**b**) ST100.

Figure 15 shows the load back electromotive force waveforms of the motors made of the two materials at 23 °C and 100 °C. It can be seen that the change degree of the back EMF of the motors made of the two materials with the temperature is different and the sinusoidal property of the back EMF of the AA motor is significantly worse with the increase in temperature, while the deterioration degree of the back EMF of the ST100 motor is smaller with the increase in temperature. This is because the AA is more sensitive to temperature than the ST100 material. With the increase in temperature, the shape of the magnetization curve of the AA changes greatly, which has an adverse effect on the back EMF of the AA motor.

**Figure 16:**
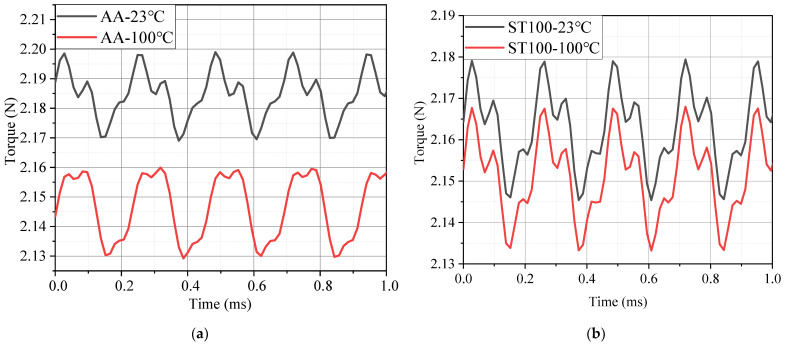
Torques of AA and ST100 motors at different temperatures: (**a**) AA; and (**b**) ST100.

Figure 16 shows the rated torque waveforms of the motors made of the two materials at 23 °C and 100 °C. It can be inferred from the test results of the magnetic properties of the two materials in Section 3 that the saturation flux density of the AA is lower than that of the ST100 silicon steel material, so the stator teeth of the AA motor are more prone to magnetic saturation phenomenon in the same motor, resulting in an increase in magnetic leakage, and ultimately, the rated torque of the AA motor is lower than that of the ST100 motor. However, the actual simulation results show that the rated torque of the AA motor is 1.01% larger than that of the ST100 motor, because the magnetic density of the teeth of the AA motor is about 1.4 T under the rated working conditions and has not reached saturation. According to the magnetization curves of the two materials measured in Section 3, at this time, under the same magnetic field strength, the AA has higher permeability, and the magnetic flux density of the AA is greater than that of the ST100, so the torque of the AA motor is larger. However, after considering the influence of temperature on the magnetic properties, the torque of motors of both materials decreased. According to the magnetic properties test data in Section 3, the magnetic flux densities of the AA and ST100 both changed with the temperature. Therefore, after considering the influence of temperature, the torque of the ST100 motor is reduced by 0.55%, and the reduction degree of the AA motor torque is as high as 1.74%, which is 3.16 times than that of the ST100 motor torque reduction. The average torque of the AA motor is reduced to 2.145 N, which is lower than the 2.151 N of the ST100 motor. Numerically, it will be found that the reduction inf the torque of the AA motor with an increasing temperature is very small in this manuscript, which is due to the low rated torque of the motor in this manuscript. However, if the torque is reduced by only 1.74% in some large torque motors, it will be a large value. If you choose to ignore the influence of the temperature, it may reduce the service life of the motor and even lead to damage. On the other hand, if the working conditions of the designed motor are those of some industrial precision instruments (such as CNC lathes), the torque variation of 1.74% may cause the machined product to fail to meet the requirements. So, it is necessary to consider the effect of temperature when designing a motor with high torque requirements, and to pay more attention to the effect of temperature when choosing an AA as a fixed rotor material. It can be further proved that the temperature sensitivity of the magnetization characteristics of the AA is greater than that of the ST100 by the change in the torque of the two materials with the temperature.

## 5. Conclusions

This paper presents an accurate electromagnetic simulation method for a high-speed permanent magnet synchronous motor (PMSM). The magnetization and iron loss characteristics of the AA and ST100 were experimentally characterized at different temperatures and different test frequencies. It can be observed from the experimental data that the temperature sensitivity of the magnetization characteristics and iron loss characteristics of the AA is significantly greater than that of the ST100.

1. The experiments in this manuscript tested the magnetic properties of the AA and ST100 under the coupling conditions of different temperature fields and different frequency fields. Compared with the traditional magnetic properties testing methods such as Epstein’s square circle method and the monolithic method, the testing method in this manuscript is more novel and the result is more accurate. When the temperature increases, the magnetic flux density of the AA decreases significantly more than that of the ST100. After the temperature increases from −40 °C to 100 °C, the magnetic flux densities of the AA and ST100 decreases by 0.6% and 10.3%, respectively. And the magnetic permeability decreases. The torque simulation results of the motor model verify that the influence of the temperature on the magnetic flux density of the AA will change the output torque of the motor. After considering the temperature effect of the actual operation of the motor, the AA motor torque is reduced by 1.74%, which is 3.16 times that of the ST100 motor. Therefore, special attention should be paid to the influence of temperature when using an AA to design a large torque motor or high torque precision motor.
2. In this manuscript, a new simulation idea based on the actual magnetic property data of the AA at different temperatures combined with multiple iterative coupled thermal simulations is proposed. In the process of motor thermal simulation, it is necessary to consider the coupling relationship between the temperature and material iron loss to achieve iterative thermal simulation. When an AA is selected as the motor stator material, it is particularly important to consider the iterative thermal simulation of the coupling relationship between the temperature and iron loss. If there is no iterative treatment in the thermal simulation process, errors of up to 11 °C or more will be generated. The iterative thermal simulation method in this paper provides an important simulation idea for the precise design of an AA motor.
3. When the temperature increases, the iron loss reduction degree of the AA is also significantly greater than that of the ST100. After the temperature increases from −40 °C to 100 °C, the iron loss reduction degree of the AA and ST100 is 5.27% and 38.37%, respectively. The separation results of the iron loss coefficient show that the iron loss coefficient of the AA and ST100 changes less with the frequency and more with the temperature, and the iron loss coefficient of the AA changes much more with the temperature than that of the ST100. The simulation results of the iron loss of the motor model verify that the iron loss of the teeth of the AA motor will be significantly reduced when the temperature rises. The more novel and detailed motor simulation ideas proposed in this manuscript can provide practical reference for the design of AA motors. In subsequent research, the sensitivity of the AA’s magnetic properties to mechanical stress will be explored, and the effect of temperature on the AA’s magnetic properties as studied in this paper will be combined to explore the variation law of the AA’s magnetic properties under the condition of multiple physical fields. Based on this, the forward design of an AA motor under the coupling effect of multiple physical fields will be carried out.

## Figures and Tables

**Table 1:** Low-frequency iron loss and high-frequency iron loss of AA and ST100 (temperature = 23 °C).

Type	Ps_50 Hz 1.0 T_	Ps_800 Hz 1.0 T_
AA	0.28 W/kg	5.81 W/kg
ST100	1.21 W/kg	30.76 W/kg

**Table 2:** Comparison of iron loss coefficient between AA and ST100 at different frequencies (temperature = 100 °C).

*f*/(Hz)	*K_h_* (AA)	*K_h_* (ST100)	*K_c_* (AA)	*K_c_* (ST100)
50	0.00344	0.01432	3.01 × 10^−6^	2.42 × 10^−4^
200	0.00388	0.01428	1.46 × 10^−6^	7.85 × 10^−5^
400	0.00436	0.01435	9.51 × 10^−7^	4.74 × 10^−5^
600	0.00469	0.01436	9.01 × 10^−7^	3.54 × 10^−5^
800	0.00502	0.01431	7.89 × 10^−7^	2.98 × 10^−5^
1000	0.00532	0.01434	7.14 × 10^−7^	2.65 × 10^−5^

**Table 3:** Comparison of iron loss coefficient between AA and ST100 at different temperatures (frequency = 50 Hz).

Temperature (°C)	*K_h_* (AA)	*K_h_* (ST100)	*K_c_* (AA)	*K_c_* (ST100)
−40	0.0036	0.01415	4.37 × 10^−5^	2.78 × 10^−4^
23	0.00364	0.01425	1.69 × 10^−5^	2.76 × 10^−4^
75	0.00351	0.01428	1.21 × 10^−5^	2.58 × 10^−4^
100	0.00344	0.01422	3.01 × 10^−6^	2.55 × 10^−4^

**Table 4:** Permanent magnet synchronous motor performance parameters.

Parameter	Value	Parameter	Value
Rated power/kW	2	Peak power/kW	3.6
Rated speed/rpm	21,000	Peak speed/rpm	25,000
DC bus voltage/V	410	Peak current/A	13

## Data Availability

The data are not publicly available for privacy reasons. The data presented in this study are available from the corresponding author.
